# Causes of Death and Characteristics of Decedents With Viral Hepatitis, United States, 2010

**DOI:** 10.1093/cid/cit642

**Published:** 2013-09-24

**Authors:** Kathleen N. Ly, Jian Xing, R. Monina Klevens, Ruth B. Jiles, Scott D. Holmberg

**Affiliations:** Division of Viral Hepatitis, Centers for Disease Control and Prevention, Atlanta, Georgia

**Keywords:** viral hepatitis, mortality, death certificates, causes of death

## Abstract

**Background.:**

Previous research indicates that the mortality burden from viral hepatitis is growing, particularly among middle-aged persons. To monitor progress toward prevention goals, it is important to continue to document characteristics and comortalities of these deaths. This study sought to examine demographic characteristics and the most frequent causes of death among decedents with a viral hepatitis–related death.

**Methods.:**

A cross-sectional study was performed on approximately 2.4 million death records from 2010. We calculated mortality rates for decedents with and without hepatitis A, B, and C virus (HAV, HBV, and HCV) and relative risks for the most frequently cited conditions in decedents with and without HBV and HCV.

**Results.:**

In 2010, there were 18 473 (0.7%) deaths with HAV, HBV, and HCV listed among causes of death, disproportionately in those aged 45–64 years. Among the 10 frequent causes of death, decedents listing HBV or HCV died, on average, 22–23 years earlier than decedents not listing these infections. HBV- and HCV-infected decedents aged 45–64 years had an increased risk of having the following conditions reported than decedents without these infections: cancer of liver and intrahepatic bile duct; fibrosis, cirrhosis, and other liver diseases; alcohol-related liver disease; gastrointestinal hemorrhage; human immunodeficiency infection; acute and unspecified renal failure; and septicemia (HCV only).

**Conclusions.:**

Decedents with other causes of death that include HBV or HCV died 22–23 years earlier than decedents not listing these infections. These data suggest and support the need for prevention, early identification, and treatment of HBV and HCV.

In the United States, hepatitis A virus (HAV), hepatitis B virus (HBV), and hepatitis C virus (HCV) are nationally notifiable infectious conditions and are routinely reported and monitored through national surveillance [1]. Hepatitis A and acute hepatitis B and C are reportable by law in all states and the District of Columbia; chronic hepatitis B and C are reportable by law in 43 states and the District of Columbia. Reporting of these conditions is based upon standard case definitions established in collaboration with the Centers for Disease Control and Prevention (CDC) and the Council of State and Territorial Epidemiologists [2].

While the incidence of hepatitis A, B, and C is at an all-time low [1]***—***creating the misconception that further prevention efforts are unnecessary***—***foodborne outbreaks for hepatitis A and healthcare-associated outbreaks for hepatitis B and C continue to occur each year [1]. For hepatitis A, mortality occurs most frequently among persons aged ≥45 years [1] and persons with underlying chronic liver disease [3]. For hepatitis B and C, mortality occurs most frequently among persons aged 45–64 years [4], and chronic infection accounts for the majority of the total burden. One reason why mortality occurs disproportionately in this relatively younger age group is because up to 65% [5] of the estimated 730 000 [6] US residents with hepatitis B and up to 75% [5] of the estimated 3.2 million [7] US residents with hepatitis C are asymptomatic; many are unaware of their infection [8]. Still, these prevalence estimates, which were obtained from the National Health and Nutrition Examination Survey, are considered conservative because high-risk groups, specifically homeless and institutionalized persons, were not included. Although important for estimating and describing the total burden of disease and for tracking and targeting prevention activities, an analysis describing hepatitis A, B, and C mortality rates by detailed demographic characteristics has not been performed for the United States.

Additionally, while hepatitis B and C are well-recognized causes of liver-related disease [9], understanding the effect of these viruses on other frequently occurring comorbidities that lead to death is becoming appreciated [10–13]. Specifically, studies have concluded that HCV infection significantly increased the risk of dying from all causes and nonliver-related causes [10, 11]. Additionally, all-cause mortality among HCV-infected patients in 4 US healthcare networks was nearly 3 times higher than all-cause mortality among HCV-uninfected persons in the noninstitutionalized US population [13]. However, similar studies for hepatitis B have not been performed in a representative US population. Additionally, to our knowledge, this is the first study to document the most common or frequent causes of death among decedents with hepatitis B and C.

Our goal was to use US multiple-cause-of-death (MCOD) data, mainly for hepatitis B and C, from 2010 to (1) characterize the national burden of mortality associated with and without hepatitis A, B, and C by describing incidence rates for select detailed demographic characteristics; (2) compare the most frequently listed causes of death among persons with and without a death associated with hepatitis B and C; and (3) calculate the risk of dying with the most frequently cited conditions among persons aged 45–64 years with a death associated with hepatitis B and C.

## METHODS

### MCOD Data

This study used the public-use 2010 US MCOD data file, which contains information on all registered deaths occurring within the calendar year [14]. State vital registration offices house these death certificates and, through a cooperative agreement, compile and share this information with the National Center for Health Statistics (NCHS). NCHS then uses this information to generate the annual national multiple-cause mortality datasets.

The conditions on the cause of death section of the death certificate are reported by the decedent’s physician, hospital residents, medical examiner, or coroner. The types of causes of death are the underlying, immediate, intermediate, and contributing causes of death that, together, are called the multiple causes of death. In MCOD files, these conditions are translated into *International Classification of Diseases, Tenth Revision* (*ICD-10*) [15], codes by highly skilled nosologists at NCHS using 2 schemes: entity axis and record axis. The entity axis represents a direct transcription of each disease entity listed on the cause of death section of the death certificate. The record axis represents a modified version of the entity axis in which repetitive conditions and inconsistencies are removed, related conditions are joined, and coding rules are followed [16]. An example of how conditions are coded is as follows: a death certificate with cirrhosis of liver and alcoholism as causes of death would be directly transcribed to *ICD-10* codes K74 (cirrhosis of liver without mention of alcohol) and F10 (alcohol dependence syndrome) as entity axis conditions. These conditions represent separate entities for the same death record. Searching for death records with *ICD-10* code K74 would, on the surface, seem that such records had no mention of alcohol. Therefore, a preferable record axis code would be K70.3 (alcoholic cirrhosis of liver), which would encompass both *ICD-10* codes K74 and F10.

### Definitions

For the purpose of this study, hepatitis A-, B-, and C-related deaths were defined using 2 definitions. First, deaths citing HAV (*ICD-10*: B15), HBV (*ICD-10*: B16, B17.0, B18.0, and B18.1), or HCV (*ICD-10*: B17.1 and B18.2) as the underlying cause or associated cause of death in the record axis was counted. Second, any death with HIV (*ICD-10*: B20–B24) as the underlying cause and HAV, HBV, or HCV as an associated cause of death in either the record or entity axis was counted. The second definition was implemented to ensure that deaths where coinfection with HIV and viral hepatitis occurred were not excluded due to the frequent reassignment of HIV as the underlying cause of death and the tendency for viral hepatitis to be excluded when translation from entity to record axis occurs [4, 18, 19]. For hepatitis B and C, the decision to combine acute and chronic *ICD-10* codes was based on a study that found that chronic hepatitis B and C deaths were often incorrectly coded as acute [20]. The term “viral hepatitis” refers to hepatitis A, B, and C, collectively. The terms “with hepatitis” and “without hepatitis” are used for decedents who had and did not have hepatitis listed as a cause of death, respectively.

### Statistical Analyses

Demographic information on age, race/ethnicity, and sex were examined, and mortality rates were calculated from this information. For this analysis (Table 1), deaths listing more than 1 hepatitis infection were assigned a single infection based on a mutually exclusive hierarchy: hepatitis A > hepatitis B > hepatitis C. The hierarchy was based on the need to fully describe characteristics of deaths with hepatitis A and B as hepatitis C carried the highest mortality burden and the degree of co-hepatitis infection was small. Age was divided into the following categories: 0–34, 35–44, 45–54, 65–74, and ≥75 years. Race/ethnicity was classified as Asian/Pacific Islander, American Indian/Alaska native, non-Hispanic (NH) white, NH black, and Hispanic. Mortality rates were calculated using the 2010 US bridged-race approximations [21] and were standardized to the age distribution of the 2000 US standard population [22]. The Poisson distribution was used to estimate the variance for rates and to calculate 95% confidence intervals (CIs) [23].

To determine the most frequently reported causes of death (Tables 2 and 3), *ICD-10* codes among deaths with and without hepatitis B and C were isolated from the record axis and classified according to the Clinical Classifications Software (CCS) for *ICD-10*; this is a well-developed categorization scheme that collapsed approximately 32 000 *ICD-10* codes into 260 clinically meaningful and manageable categories [24]. For the CCS category labeled “Other Liver Diseases” in decedents with hepatitis B or hepatitis C, liver fibrosis and cirrhosis (*ICD-10*, K74) was the most frequently listed; therefore, we renamed this category with the more descriptive label of “Fibrosis, Cirrhosis, and Other Liver Diseases.” Liver-associated conditions included the following CCS categories: fibrosis, cirrhosis, and other liver diseases; other hepatitis infections; cancer of the liver and intrahepatic bile duct; alcohol-related liver disease; and gastrointestinal hemorrhage. The median age at death for each of the top 10 CCS categories was examined. Then, the difference was determined by calculating the average of the median ages for the 10 most frequently cited causes among decedents with and without hepatitis B and C and subtracting these 2 averages.

Relative risks were calculated to quantify the risk of dying with the 15 most frequently cited conditions among decedents aged 45–64 years who had hepatitis B and C listed among causes of death. The comparison group was decedents belonging to the same age group who did not have hepatitis B and C listed. For this analysis, we included deaths with either hepatitis B alone or hepatitis C alone (but not those who were coinfected) to remove the effect of potential confounders on the results of deaths with hepatitis B, which accounted for 30% of hepatitis B deaths. We separately compared the relative risks of all deaths with hepatitis B together (hepatitis B alone plus hepatitis B/C coinfected) with hepatitis B alone and found that the magnitude of association of liver-associated and HIV-associated conditions with hepatitis B would be more similar to those of hepatitis C had hepatitis B/C coinfected deaths been included, therefore justifying the removal of the coinfected.

The 95% CIs were used to determine the variance and statistical significance of each relative risk estimate. Data were analyzed using SAS software, version 9.2 (SAS Institute, Cary, NC).

## RESULTS

**Figure 1.**
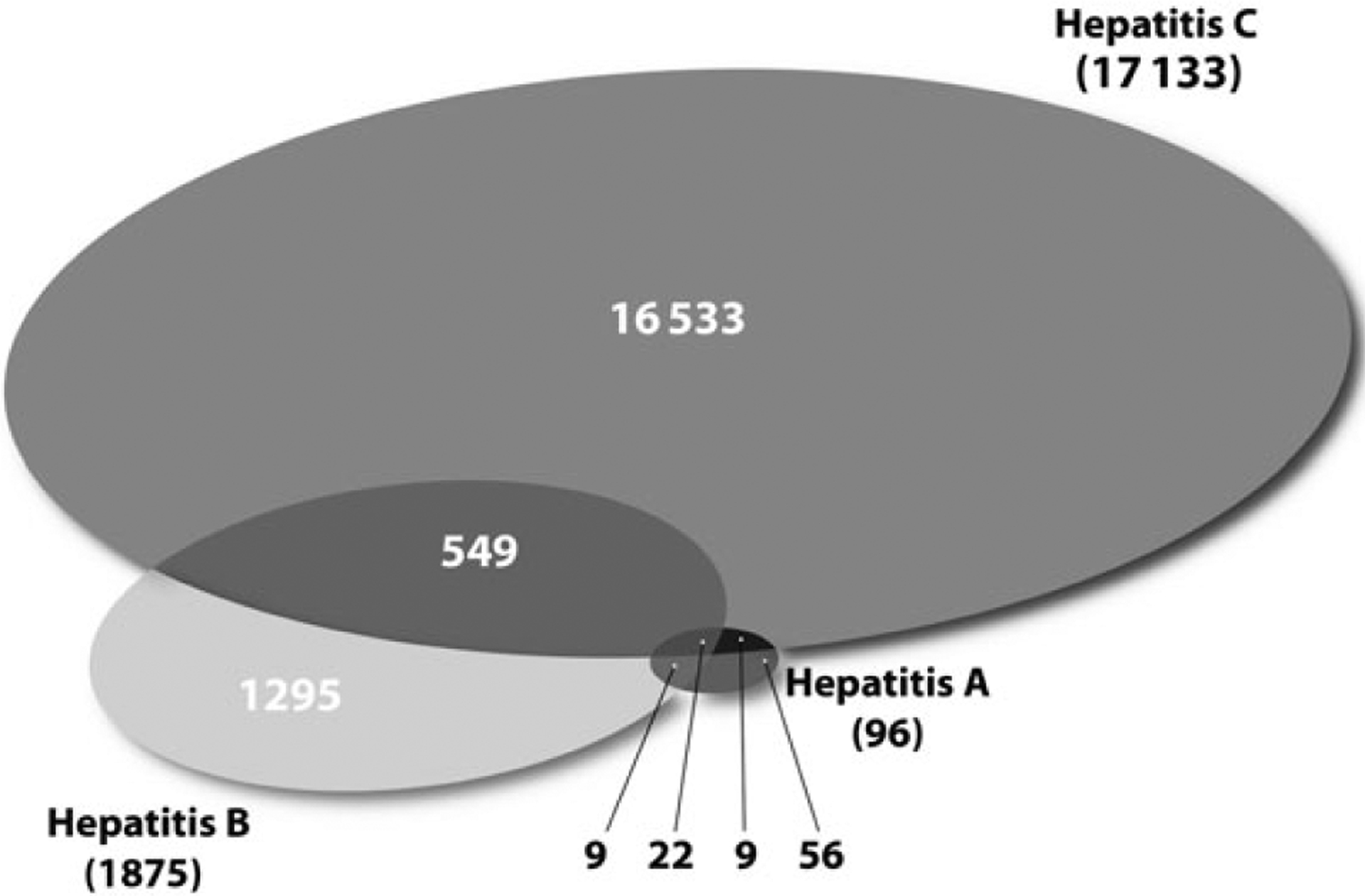
Number of deaths reported with hepatitis A, B, and C in the United States, 2010, N = 18 473.

In 2010, 2 472 542 deaths were registered in the United States, of which 18 473 (0.7%) listed hepatitis A, B, or C among causes of death. This implied an age-adjusted mortality rate of 5.2 deaths per 100 000 population (Table 1). Hepatitis A was listed as the underlying cause of 30 deaths (<0.01%) and as any cause of 96 deaths (<0.01%; Figure 1). Hepatitis B was listed as the underlying cause of 589 deaths (0.02%) and as any cause of 1875 deaths (0.08%). Hepatitis C was listed as the underlying cause of 6857 deaths (0.28%) and as any cause of 17 113 deaths (0.69%). Among deaths with hepatitis A, B, or C, 92.6% were with hepatitis C, 10.1% were with hepatitis B, and 0.5% was with hepatitis A. Among deaths with hepatitis C, 3.4% had hepatitis A and/or B. Among deaths with hepatitis B, 1.7% had hepatitis A.

Among decedents with hepatitis A, the highest mortality rates were observed among persons aged ≥45 years and accounted for 89.6% of hepatitis A–related deaths (Table 1). Among decedents with hepatitis B, the highest mortality rates were observed among persons aged 55–64 years, Asians/Pacific Islanders, and males. Among decedents with hepatitis C, the highest mortality rates were observed among persons aged 55–64 years; the American Indian/Alaskan Native, NH black, and Hispanic race/ethnic groups; and males. In comparison, among decedents without viral hepatitis, the highest mortality rates were observed among persons aged ≥75 years, NH blacks, and males.

The most frequently listed category among deaths with either hepatitis B or hepatitis C was fibrosis, cirrhosis, and other liver diseases (45.3% and 48.4%, respectively; Tables 2 and 3). Cancer of the liver, including hepatocellular carcinoma, and intrahepatic bile duct and alcohol-related liver disease were also frequently reported in deaths with hepatitis B and C (11.6%–22.8%). The most frequently reported nonliver-associated conditions among decedents with either hepatitis B or C were cardiac arrest and ventricular fibrillation, substance-related mental disorders, diabetes mellitus without complication, essential hypertension, and adult respiratory failure insufficiency arrest (8.9%–14.0%). Septicemia (except in labor) was among the top 10 most frequently reported conditions for hepatitis C but not for hepatitis B.

For deaths without hepatitis B or C, liver-associated conditions were not among the top 10 categories. Further, the top causes occurred at a lower frequency for any 1 category (7.2%–15.1%) than the top causes among deaths with hepatitis B or C (8.9%–48.4%; Tables 2 and 3). Among the 10 most frequently cited causes, deaths listing hepatitis B and C occurred at an average median age of 22–23 years younger than deaths not listing hepatitis B and C.

Among decedents aged 45–64 years, the relative risks for conditions associated with a significantly increased risk of dying with hepatitis B ranged from 14.0 (cancer of liver and intrahepatic bile duct) to 2.6 (acute and unspecified renal failure; Table 4). Coronary atherosclerosis and other heart disease and substance-related mental disorders (relative risk, 0.5 and 0.7, respectively) were the 2 conditions associated with a significantly decreased risk of dying with hepatitis B.

Among the same age group, the relative risks for conditions associated with a significantly increased risk of dying with hepatitis C ranged from 12.0 (cancer of liver and intrahepatic bile duct) to 1.5 (septicemia, except in labor; Table 5). The relative risks for conditions associated with a significantly decreased risk of dying with hepatitis C ranged from 0.6 (all external causes of injury and poisoning) to 0.9 (respiratory failure, insufficiency, arrest***—***adult).

## DISCUSSION

Our analysis of 2010 MCOD data identified 18 473 deaths reported with viral hepatitis. If ranked as a leading cause of death using NCHS’s list [25], viral hepatitis would rank as the 15th leading cause of death.

Hepatitis C alone was identified as a cause of nearly 90% of these deaths; the majority of those occurred in persons aged 45–64 years. The disproportionate burden in this age group is consistent with other studies [4, 7, 18]. As a result, in 2012, the CDC recommended 1-time hepatitis C testing for persons born during 1945–1965 (aged 45–65 years in 2010) [26], especially because birth-cohort screening in primary care settings is cost effective [27]. Our study provides evidence that strengthens this national recommendation. Moreover, the well-demonstrated increased mortality of both liver-associated and nonliver-associated conditions in HBV- and HCV-infected decedents provides key evidence to get more people treated before they develop serious illness.

In our comparative cause-of-death analysis, hepatitis B- or C-related deaths most frequently also had liver-associated conditions, which supports the established literature on outcomes of chronic hepatitis infection. Our most significant finding, however, was that among decedents with the same cause of death, persons with hepatitis B or C died about 2 decades younger than persons without these infections. Prevention and early treatment of hepatitis B and C will help prevent these early deaths. Evidence suggests that early therapeutic intervention improves all-cause hepatitis B and C mortality [28–30].

Illicit drug use is associated with an increased likelihood of HBV and HCV infection [7]. In this study, while we showed that substance-related mental disorders were reported frequently among causes of death in persons aged 45–64 years, regardless if HBV and HCV infections were present, the relative risk of dying with a substance-related mental disorder, however, was less likely in deaths with hepatitis B and was not significant in deaths with hepatitis C. For hepatitis B, the decreased likelihood of dying may be explained by the fact that the majority of HBV-infected decedents in the 45–64 year age group were Asians/Pacific Islanders, who most likely acquired their infection during birth or early childhood. For hepatitis C, the insignificant relative risk of dying may be explained by the fact that most persons aged 45–64 years may have used illicit drugs infrequently during their youth, and the behavior is more common among adolescents and young adults. To support this assumption, we did find a significant elevated risk of dying with substance-related mental disorders in HCV-infected decedents aged 0–44 years (data not shown).

Our study demonstrated racial/ethnic disparities in deaths with hepatitis C, specifically among American Indians/Alaskan Natives, NH blacks, and Hispanics. While health disparities in minorities with chronic HCV infection have been documented since as early as 1999 [4, 31, 32], we and others [4] showed that this trend, unfortunately, has not improved.

Although we could not obtain vaccination status from death certificates, our data showed that 3.4% of decedents with hepatitis C had hepatitis A and/or hepatitis B and 1.7% of decedents with hepatitis B had hepatitis A, indicating vaccination was probably not received. Even though hepatitis A and B vaccination is recommended for HCV-infected patients [33], this recommendation had the lowest quality-of-care indicator score in an evaluation study of HCV-infected patients***—***only 22% received hepatitis A vaccination and only 26% received hepatitis B vaccination or had documented immunity [34].

Although the results of this study are population based, the findings should be interpreted with caution. First, the inaccuracy of cause-of-death coding on death certificates is a significant problem that can lead to underestimates in the viral hepatitis mortality burden [35, 36]; therefore, the hepatitis death estimates in this analysis likely represent only a small fraction of the true burden. Despite having guidelines and training in place, a study at Johns Hopkins Medical Institutions found that more than 40% of causes of death were improperly filled out [36]. In addition to the variability in completeness of recording viral hepatitis deaths, there are data from studies that used medical records to validate hepatitis B and/or C as causes of death in healthcare networks (Mahajan et al, unpublished data) [37]. Investigators found that for patients who had a known HBV [37] or HCV [37, 38] infection associated with chronic liver disease at death, this information was often not reported on death certificates, even when end stage liver disease or hepatocellular carcinoma were listed as the main cause of death. The viral hepatitis mortality burden is even further underestimated by undiagnosed hepatitis infections and deaths unrelated to the decedent’s hepatitis infection, such as those that resulted from a suicide or vehicle accident. Because death certificate data imperfectly collect cause of death information, this analysis can only provide data for which viral hepatitis is or is not mentioned on the death certificate. Despite these limitations, MCOD data are invaluable in that they capture all registered deaths in the United States, providing an insightful view into the national burden of viral hepatitis mortality.

In summary, viral hepatitis was listed as a cause of more than 18 000 recorded US deaths in 2010, and there are many who are likely not diagnosed or recorded as having these hepatitides [37]. Because these data demonstrated that death occurred 22–23 years earlier among persons with an HBV- or HCV-related death, prevention efforts should be expanded to further (1) promote hepatitis A and B vaccination among recommended target groups, (2) increase hepatitis B and C screening to get more people into care and earlier treatment, and (3) treat alcohol- and drug-related disorders [26].

## Figures and Tables

**Table 1.** Age-Adjusted and Age-Specific Mortality Rates With and Without Hepatitis A, B, and C by Demographic Characteristics in the United States, 2010

Characteristic	Hepatitis A, B, or C			Hepatitis A			Hepatitis B			Hepatitis C			Non-Hepatitis A, B, or C		
	Number	Rate	(95% CI)	Number	Rate	(95% CI)	Number	Rate	(95% CI)	Number	Rate	(95% CI)	Number	Rate	(95% CI)
Overall	18 473	5.19	(5.11–5.28)	96	0.03	(.02–.03)	1844	0.54	(.51–.57)	16 533	4.62	(4.54–4.71)	2 454 069	764.76	(763.72–765.79)
Age group, y^a^															
≤34	172	0.12	(.10–.14)	3	0	(.00–.01)	50	0.03	(.03–.05)	119	0.08	(.07–.10)	106 344	72.88	(72.44–73.32)
35–44	894	2.18	(2.04–2.32)	7	0.02	(.01–.04)	157	0.38	(.33–.45)	730	1.78	(1.65–1.91)	69 421	169.03	(167.78–170.29)
45–54	5690	12.64	(12.32–12.98)	25	0.06	(.04–.08)	475	1.06	(.96–1.15)	5190	11.53	(11.22–11.85)	178 093	395.70	(393.87–397.55)
55–64	7996	21.92	(21.44–22.40)	35	0.1	(.07–.13)	618	1.69	(1.57–1.83)	7343	20.13	(19.67–20.59)	303 545	832.02	(829.07–834.99)
65–74	2180	10.04	(9.63–10.47)	10	0.05	(.02–.09)	297	1.37	(1.22–1.53)	1873	8.63	(8.24–9.03)	405 790	1868.84	(1863.10–1874.60)
≥75	1539	8.29	(7.89–8.72)	16	0.09	(.05–.14)	247	1.33	(1.18–1.51)	1276	6.88	(6.51–7.26)	1 390 539	7494.33	(7481.88–7506.79)
Race/Ethnicity^b^															
White, NH	11 342	4.34	(4.26–4.42)	65	0.03	(.02–.03)	868	0.34	(.32–.37)	10 409	3.97	(3.89–4.04)	1 960 321	778.83	(777.78–779.87)
Black, NH	3443	8.93	(8.81–9.04)	16	0.04	(.03–.05)	385	1.02	(.98–1.06)	3042	7.87	(7.76–7.97)	280 358	917.89	(916.76–919.03)
Hispanic	2503	7.38	(7.28–7.48)	12	0.03	(.03–.04)	143	0.45	(.42–.47)	2348	6.9	(6.80–7.00)	141 470	541.91	(541.04–542.78)
Asian/Pacific Islander	842	6.11	(6.02–6.20)	2	0.02	(.01 −.02)	425	2.98	(2.91–3.04)	415	3.11	(3.05–3.18)	50 707	426.76	(425.99–427.53)
American Indian/Alaska Native	270	10.85	(10.73–10.97)	1	0.05	(.04–.06)	17	0.73	(.70–.77)	252	10.07	(9.95–10.19)	15 314	835.62	(834.54–836.70)
Sex															
Male	13 132	7.65	(7.55–7.76)	74	0.05	(.04–.06)	1359	0.83	(.80–.87)	11 699	6.77	(6.67–6.87)	1 222 070	875.24	(874.13–876.34)
Female	5341	2.92	(2.85–2.98)	22	0.01	(.01 −.02)	485	0.27	(.26–.29)	4834	2.63	(2.57–2.69)	1 231 999	671.73	(670.76–672.70)

Source: Centers for Disease Control and Prevention. National Center for Health Statistics. National Vital Statistics System.

Mortality rates are presented as per 100 000 population. Deaths listing >1 type of hepatitis infection were classified by using the following mutually exclusive hierarchy: hepatitis A > hepatitis B > hepatitis C. Abbreviations: CI, confidence interval; NH, non-Hispanic.

a Two deaths for hepatitis C were not represented under the age group category due to missing data.

b Six deaths for hepatitis B, 67 deaths for hepatitis C, and 5899 deaths for non–hepatitis A, B, or C were not represented under the race/ethnicity category due to missing data.

**Table 2.** Most Frequently Listed Categories of Causes of Death and Median Age at Death Among Decedents With and Without Hepatitis B in the United States, 2010

Ranking	Category	No.	%^a^	Median Age, y
Deaths with hepatitis B				
1	CCS 151: Fibrosis, cirrhosis, and other liver diseases	850	45.3	58
2	CCS 6: Hepatitis (excluding hepatitis B)	573	30.6	56
3	CCS 16: Cancer of liver and intrahepatic bile duct	427	22.8	59
4	CCS 107: Cardiac arrest and ventricular fibrillation	232	12.4	58
5	CCS 67: Substance-related mental disorders	218	11.6	57
6	CCS 150: Liver disease, alcohol-related	211	11.3	55
7	CCS 157: Acute and unspecified renal failure	198	10.6	57
8	CCS 49: Diabetes mellitus without complication	190	10.1	59
9	CCS 98: Essential hypertension	169	9.0	61
10	CCS 131: Respiratory failure, insufficiency, arrest (adult)	168	9.0	58
Deaths without hepatitis B				
1	CCS 101: Coronary atherosclerosis and other heart disease	372 568	15.1	82
2	CCS 107: Cardiac arrest and ventricular fibrillation	339 188	13.7	79
3	CCS 68: Senility and organic mental disorders	332 865	13.5	87
4	CCS 98: Essential hypertension	297 406	12.0	82
5	CCS 108: Congestive heart failure, nonhypertensive	279 188	11.3	85
6	CCS 131: Respiratory failure, insufficiency, arrest (adult)	269 112	10.9	78
7	CCS 127: Chronic obstructive pulmonary disease and bronchiecstasis	262 622	10.6	78
8	CCS 67: Substance-related mental disorders	251 286	10.2	71
9	CCS 49: Diabetes mellitus without complication	205 112	8.3	76
10	CCS 109: Acute cerebrovascular disease	177 231	7.2	82

Source: Centers for Disease Control and Prevention. National Center for Health Statistics. National Vital Statistics System.

Abbreviation: CCS, Clinical Classifications Software.

a Cumulative percentages will not equal 100% because a death listing multiple causes of death can be placed into >1 category and only the 10 most frequent categories among deaths with hepatitis B are listed. The denominator equals 1875 for deaths with hepatitis B and 2 470 667 for deaths without hepatitis B.

**Table 3.** Most Frequently Listed Categories of Causes of Death and Median Age at Death Among Decedents With and Without Hepatitis C in the United States, 2010

Ranking	CCS Category	No.	%^a^	Median Age
Deaths with hepatitis C				
1	CCS 151: Fibrosis, cirrhosis, and other liver diseases	8278	48.4	57
2	CCS 16: Cancer of liver and intrahepatic bile duct	2900	17.0	59
3	CCS 150: Liver disease, alcohol-related	2831	16.5	55
4	CCS 67: Substance-related mental disorders	2390	14.0	56
5	CCS 107: Cardiac arrest and ventricular fibrillation	1817	10.6	57
6	CCS 49: Diabetes mellitus without complication	1741	10.2	58
7	CCS 98: Essential hypertension	1683	9.8	59
8	CCS 2: Septicemia (except in labor)	1658	9.7	56
9	CCS 131: Respiratory failure, insufficiency, arrest (adult)	1565	9.2	57
10	CCS 157: Acute and unspecified renal failure	1523	8.9	57
Deaths without hepatitis C				
1	CCS 101: Coronary atherosclerosis and other heart disease	371 852	15.1	82
2	CCS 107: Cardiac arrest and ventricular fibrillation	337 603	13.8	79
3	CCS 68: Senility and organic mental disorders	332 694	13.6	87
4	CCS 98: Essential hypertension	295 892	12.1	82
5	CCS 108: Congestive heart failure, nonhypertensive	278 617	11.4	85
6	CCS 131: Respiratory failure, insufficiency, arrest (adult)	267 715	10.9	78
7	CCS 127: Chronic obstructive pulmonary disease and bronchiecstasis	261 400	10.7	78
8	CCS 67: Substance-related mental disorders	249 114	10.2	71
9	CCS 49: Diabetes mellitus without complication	203 561	8.3	76
10	CCS 109: Acute cerebrovascular disease	176 844	7.2	82

Source: Centers for Disease Control and Prevention. National Center for Health Statistics. National Vital Statistics System.

Abbreviation: CCS, Clinical Classifications Software.

a Cumulative percentages will not equal 100% because a death listing multiple causes of death can be placed into >1 category and only the 10 most frequent categories among deaths with hepatitis C are listed. The denominator equals 17 113 for deaths with hepatitis C and 2 455 429 for deaths without hepatitis C.

**Table 4.** Most Frequently Listed Categories of Causes of Death Among Decedents Aged 45–64 Years With and Without Hepatitis B in the United States, 2010

Ranking by Frequency	Category	With/Without CCS Category	Hepatitis B^a^		RR (95% CI)
			With n = 662	Without n = 494 662	
1	CCS 151: Fibrosis, cirrhosis, and other liver diseases	With	299 (45.2%)	33 208 (6.7%)	6.73 (6.18, 7.32)
		Without	363 (54.8%)	461 454 (93.3%)	
2	CCS 16: Cancer of liver and intrahepatic bile duct	With	170 (25.7%)	8855 (1.8%)	14.35 (12.58, 16.36)
		Without	492 (74.3%)	485 807 (98.2%)	
3	CCS 107: Cardiac arrest and ventricular fibrillation	With	87 (13.1%)	64 331 (13.0%)	1.01 (.83, 1.23)
		Without	575 (86.9%)	430 331 (87.0%)	
4	CCS 157: Acute and unspecified renal failure	With	79 (11.9%)	22 518 (4.6%)	2.62 (2.13, 3.23)
		Without	583 (88.1%)	472 144 (95.4%)	
5	CCS 150: Liver disease, alcohol-related	With	71 (10.7%)	14 252 (2.9%)	3.72 (2.99, 4.64)
		Without	591 (89.3%)	480 410 (97.1%)	
6	CCS 49: Diabetes mellitus without complication	With	66 (10.0%)	45 219 (9.1%)	1.09 (.87, 1.37)
		Without	596 (90.0%)	449 443 (90.9%)	
7	CCS 67: Substance-related mental disorders	With	64 (9.7%)	71 892 (14.5%)	0.67 (.53, .84)
		Without	598 (90.3%)	422 770 (85.5%)	
8	CCS 131: Respiratory failure, insufficiency, arrest (adult)	With	61 (9.2%)	49 713 (10.0%)	0.92 (.72, 1.16)
		Without	601 (90.8%)	444 949 (90.0%)	
9	CCS 5: HIV infection	With	57 (8.6%)	6082 (1.2%)	7.00 (5.46, 8.99)
		Without	605 (91.4%)	488 580 (98.8%)	
10	CCS 2: Septicemia (except in labor)	With	56 (8.5%)	34 659 (7.0%)	1.21 (.94, 1.55)
		Without	606 (91.5%)	460 003 (93.0%)	
11	CCS 259: Residual codes, unclassified	With	55 (8.3%)	18 325 (3.7%)	2.24 (1.74, 2.89)
		Without	607 (91.7%)	476 337 (96.3%)	
12	CCS 98: Essential hypertension	With	50 (7.6%)	43 621 (8.8%)	0.86 (.66, 1.12)
		Without	612 (92.4%)	451 041 (91.2%)	
13	CCS 127: Chronic obstructive pulmonary disease and bronchiectasis	With	44 (6.6%)	39 406 (8.0%)	0.83 (.63, 1.11)
		Without	618 (93.4%)	455 256 (92.0%)	
14	CCS 153: Gastrointestinal hemorrhage	With	35 (5.3%)	8334 (1.7%)	3.14 (2.27, 4.34)
		Without	627 (94.7%)	486 328 (98.3%)	
15	CCS 101: Coronary atherosclerosis and other heart disease	With	33 (5.0%)	51 532 (10.4%)	0.48 (.34, .67)
		Without	629 (95.0%)	443 130 (89.6%)	

Source: Centers for Disease Control and Prevention. National Center for Health Statistics. National Vital Statistics System.

Data are presented as No. (%) unless otherwise specified.

Abbreviations: CCS, Clinical Classifications Software; CI, confidence interval; HIV, human immunodeficiency virus; RR, relative risk.

a Identified in the absence of hepatitis C.

**Table 5.** Most Frequently Listed Categories of Causes of Death Among Decedents Aged 45–64 Years With and Without Hepatitis C in the United States, 2010

Ranking by Frequency	Category	With/Without CCS Category	Hepatitis C^a^		RR (95% CI)
			With n = 12 533	Without n = 482 791	
1	CCS 151: Fibrosis, cirrhosis, and other liver diseases	With	6009 (47.9%)	27498 (5.7%)	8.42 (8.24, 8.60)
		Without	6524 (52.1%)	455 293 (94.3%)	
2	CCS 150: Liver disease, alcohol-related	With	2375 (18.9%)	11 948 (2.5%)	7.66 (7.35, 7.97)
		Without	10 158 (81.1%)	470 843 (97.5%)	
3	CCS 16: Cancer of liver and intrahepatic bile duct	With	2138 (17.1%)	6887 (1.4%)	11.96 (11.43, 12.51)
		Without	10 395 (82.9%)	475 904 (98.6%)	
4	CCS 67: Substance-related mental disorders	With	1881 (15.0%)	70 075 (14.5%)	1.03 (.99, 1.08)
		Without	10 652 (85.0%)	412 716 (85.5%)	
5	CCS 2: Septicemia (except in labor)	With	1266 (10.1%)	33 449 (6.9%)	1.46 (1.38, 1.54)
		Without	11 267 (89.9%)	449 342 (93.1%)	
6	CCS 107: Cardiac arrest and ventricular fibrillation	With	1251 (10.0%)	63 167 (13.1%)	0.76 (.72, .80)
		Without	11 282 (90.0%)	419 624 (86.9%)	
7	CCS 49: Diabetes mellitus without complication	With	1206 (9.6%)	44 079 (9.1%)	1.05 (1.00, 1.11)
		Without	11 327 (90.4%)	438 712 (90.9%)	
8	CCS 131: Respiratory failure, insufficiency, arrest (adult)	With	1146 (9.1%)	48 628 (10.1%)	0.91 (.86, .96)
		Without	11 387 (90.9%)	434 163 (89.9%)	
9	CCS 98: Essential hypertension	With	1140 (9.1%)	42 531 (8.9%)	1.03 (.98, 1.09)
		Without	11 393 (90.9%)	440 260 (91.2%)	
10	CCS 157: Acute and unspecified renal failure	With	1093 (8.7%)	21 504 (4.5%)	1.96 (1.85, 2.08)
		Without	11 440 (91.3%)	461 287 (95.5%)	
11	CCS 127: Chronic obstructive pulmonary disease and bronchiectasis	With	967 (7.7%)	38 483 (8.0%)	0.97 (.91, 1.03)
		Without	11 566 (92.3%)	444 308 (92.0%)	
12	CCS 153: Gastrointestinal hemorrhage	With	863 (6.9%)	7506 (1.6%)	4.43 (4.14, 4.74)
		Without	11 670 (93.1%)	475 285 (98.4%)	
13	CCS 259: Residual codes, unclassified	With	856 (6.8%)	17 524 (3.6%)	1.88 (1.76, 2.01)
		Without	11 677 (93.2%)	465 267 (96.4%)	
14	CCS 5: HIV infection	With	641 (5.1%)	5498 (1.1%)	4.49 (4.15, 4.86)
		Without	11 892 (94.9%)	477 293 (98.9%)	
15	CCS 260: E codes: all (external causes of injury and poisoning)	With	579 (4.6%)	40 895 (8.5%)	0.55 (.50, .59)
		Without	11 954 (95.4%)	441 896 (91.5%)	

Source: Centers for Disease Control and Prevention. National Center for Health Statistics. National Vital Statistics System.

Data are presented as No. (%) unless otherwise specified.

Abbreviations: CCS, Clinical Classifications Software; CI, confidence interval; HIV, human immunodeficiency virus; RR, relative risk.

a Identified in the absence of hepatitis B.
